# Supplementary material for: The phased pan-genome of tetraploid European potato
Source: Nature. 2025 Apr 16;642(8067):389–97. doi: 10.1038/s41586-025-08843-0 (PMC12158759; doi:10.1038/s41586-025-08843-0)
Supplement: Supplementary file 5 — Supplementary Figs. 3–14, 20, 21, 23–27 and 34–44. [file 41586_2025_8843_MOESM5_ESM.zip › suppl_figure_20/suppl_figure_20b_chr02.pdf]

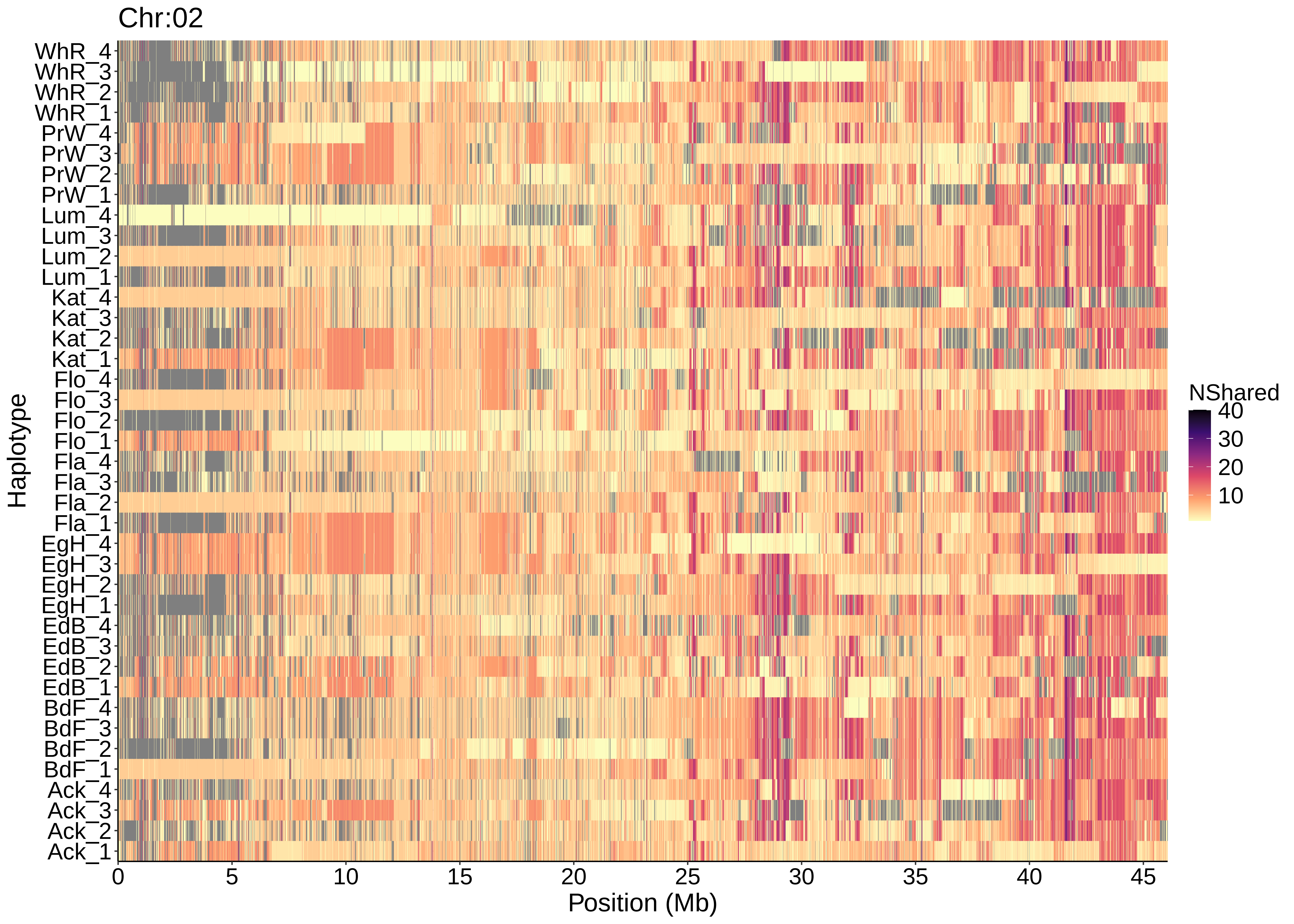

Chr:02
Haplotype
WhR_4
WhR_3
WhR_2
WhR_1
PrW_4
PrW_3
PrW_2
PrW_1
Lum_4
Lum_3
Lum_2
Lum_1
Kat_4
Kat_3
Kat_2
Kat_1
Flo_4
Flo_3
Flo_2
Flo_1
Fla_4
Fla_3
Fla_2
Fla_1
EgH_4
EgH_3
EgH_2
EgH_1
EdB_4
EdB_3
EdB_2
EdB_1
BdF_4
BdF_3
BdF_2
BdF_1
Ack_4
Ack_3
Ack_2
Ack_1
0
5
10
15
20
25
30
35
40
45
Position (Mb)
NShared
40
30
20
10
